# Cerebrospinal fluid neurofilament light levels in CLN2 disease patients treated with enzyme replacement therapy normalise after two years on treatment

**DOI:** 10.12688/f1000research.54556.1

**Published:** 2021-07-20

**Authors:** Katharina Iwan, Nina Patel, Amanda Heslegrave, Mina Borisova, Laura Lee, Rebecca Bower, Sara E. Mole, Philippa B. Mills, Henrik Zetterberg, Kevin Mills, Paul Gissen, Wendy E. Heywood

**Affiliations:** 1 UCL Institute of Child Health, University College London, London, WC1N 1EH, UK; 2 NIHR Great Ormond Street Hospital Biomedical Research Centre, University College London, London, WC1N 1EH, UK; 3 UK Dementia Research Institute, University College London, London, WC1E 6BT, UK; 4 Great Ormond Street Children's Hospital NHS Foundation Trust, London, WC1N 3JH, UK; 5 Institute of Neuroscience and Physiology, Sahlgrenska Academy at the University of Gothenburg, Gothenburg, Sweden

**Keywords:** Neuronal Ceroid lipofuscinosis, Enzyme replacment therapy, Neurofilament light

## Abstract

Classic late infantile neuronal ceroid lipofuscinosis (CLN2 disease) is caused by a deficiency of tripeptidyl-peptidase-1. In 2017, the first CLN2 enzyme replacement therapy (ERT) cerliponase alfa (Brineura) was approved by the FDA and EMA. The CLN2 disease clinical rating scale (CLN2 CRS) was developed to monitor loss of motor function, language and vision as well as frequency of generalised tonic clonic seizures. Using CLN2 CRS in an open label clinical trial it was shown that Brineura slowed down the progression of CLN2 symptoms.

Neurofilament light chain (NfL) is a protein highly expressed in myelinated axons. An increase of cerebrospinal fluid (CSF) and blood NfL is found in a variety of neuroinflammatory, neurodegenerative, traumatic, and cerebrovascular diseases. We analysed CSF NfL in CLN2 patients treated with Brineura to establish whether it can be used as a possible biomarker of response to therapy. Newly diagnosed patients had CSF samples collected and analysed at first treatment dose and up to 12 weeks post-treatment to look at acute changes. Patients on a compassionate use programme who were already receiving ERT for approximately 1yr had CSF samples collected and NfL analysed over the following 1.3 years (2.3 years post-initiation of ERT) to look at long-term changes.

All newly diagnosed patients we investigated with classical late infantile phenotype had high NfL levels >2000 pg/ml at start of treatment. No significant change was observed in NfL up to 12 weeks post-treatment. After one year of ERT, two out of six patients still had high NfL levels, but all patients showed a continued decrease, and all had low NfL levels after two years on ERT. NfL levels appear to correspond and predict improved clinical status of patients on ERT and could be useful as a biomarker to monitor neurodegeneration and verify disease modification in CLN2 patients on ERT.

## Introduction

Classic late infantile neuronal ceroid lipofuscinosis (CLN2 disease) is the second most common type of neuronal ceroid lipofuscinosis (NCL), a group of inherited progressive neurodegenerative diseases in children. ^1^ The classical form of CLN2 disease presents in most patients with early language delay followed by onset of seizures at around three years of age, ataxia, motor and cognitive decline, loss of vision, with death by early adolescence. Atypical forms of CLN2 exist, where patients may present at an older age and with much slower pace of neurodegeneration. ^2^ It is estimated that atypical patients constitute 10-20% of the total CLN2 patient cohort, although this may vary in different populations.

In the UK, 5-6 children are diagnosed with CLN2 disease each year, and it is estimated that 30-50 children are currently living with the disease. ^3^ CLN2 disease is caused by mutations in the tripeptidyl peptidase 1 ( *TPP1*) gene, which result in either loss or deficiency of the TPP1 lysosomal hydrolase. TPP1 deficiency leads to the characteristic autofluorescent neuronal ceroid lipofuscin accumulation of NCL. ^4^

Brineura (cerliponase alfa) is a human recombinant form of TPP1 enzyme replacement therapy (ERT) that was approved for treatment in the US and EU in 2017. It is administered as an intracerebroventricular infusion every two weeks. ^5^ The effect of ERT on functional decline can be measured using the CLN2 Disease Clinical Rating Scale (CLN2 CRS). ^6^ Patients on ERT are less likely to have an unreversed two-point decline in a combined motor and language function score when compared to untreated patients. ^5^ Currently the only way to monitor treatment is through the CLN2 CRS as there are no biomarkers used to monitor neurodegeneration. In this study, we measured neurofilament light (NfL) a known biomarker of neuroaxonal damage and degeneration ^7^ in patients undergoing ERT either in the initial 18 weeks of treatment or, in another cohort of patients, who were receiving ERT for at least one year, in whom we monitored NfL over the following 12-18 month period. This allowed us to determine the timing of the response of NfL levels in patients on ERT.

## Methods

### Ethical statement

The collection of samples for this study has ethical approval (13/LO/0168; IRAS ID 95005; London-Bloomsbury Research Ethics Committee) and Health Research Authority (HRA) approval and all participants provided informed written consent to participate. The study was conducted between May 2019 to October 2020 at Great Ormond Street hospital, UCL Institute of Child Health and UCL Dementia Research Institute.

### Samples

All patients were receiving intraventricular infusion of Brineura (cerliponase alfa) from BioMarin pharmaceutical. ^8^ Samples were collected for two groups of patients. A short-term/acute change (n = 5) group consisted of samples from patients at first dose of ERT and followed from 20-80 days after the start of treatment. A long-term group (n = 6) consisted of samples collected from patients who had been receiving ERT for approximately one year. Samples were collected over an additional 12-18 month period from one year of ERT. CLN2 score was performed as in Schulz et al, 2018. ^5^ The lowest initial combined CLN2 score was 2 in the short-term group and 1 in the long-term group. All patients receiving Brineura infusions at Great Ormond Street Hospital were included into the study provided the families signed an informed consent form.

Ventricular CSF samples were acquired from surplus material taken for routine infection monitoring. Collected CSF was frozen at -80°C within 24 hours of collection. CSF NfL protein concentration was measured on a Simoa HD-X analyser using the Simoa NF-light Advantage assay kit (Quanterix, Billerica, MA) after being diluted 100×, as per manufacturer’s instructions. The measurements were performed in one round of experiments using one batch of reagents with the analyst blinded to clinical data. Intra-assay coefficients of variation, monitored using internal quality control samples, were 0.3-8.9%.

### Analysis

Results were exported to Microsoft Excel ^15^ and data analysed using GraphPad Prism v 6 for statistical analysis. Non-parametric paired t-test was applied.

## Results and discussion

NfL had first been suggested as a biomarker for future treatment-monitoring of CLN3 disease as elevated CSF (2096 ± 1202 pg/ml) has been observed in patients compared to controls (345 ± 610 pg/ml). ^9^ Serum NfL concentration in CLN2 disease has been described to decrease with treatment in canine models and paediatric patients where levels were 48-fold higher than controls pre-treatment but decreased by 50% each year over more than three years of treatment. CSF NfL was monitored for the canine model and observed to correlate with serum NfL; however, the degree of change of NfL with disease progression was observed to be greater in CSF (by 100%) than in serum (56%). ^10^ A healthy paediatric NfL range has so far not been defined. NfL is observed to increase with age with the lowest range observed for 20-25 year olds as <300 pg/ml. ^11^ Therefore, values above 300 pg/ml are likely pathological.

We describe here the analysis of two separate patient cohorts collected to assess short-term change (n = 5) and long-term change (n = 6) in clinical parameters and CSF NfL over 1-2 years after the first infusion of cerliponase alfa. We observed levels of NfL at the first infusion/baseline over 2000 pg/ml for all patients. Patient information is given in Table 1.

**Table 1.** Patient clinical information.

Patient	Sex	Age at start of ERT (years)	CLN2 language and motor score	Days from first ERT to first Nfl measurement	CLN2 L+M score after most recent NFL measurement	Genetics allele 1	Genetics allele 2	Disease subtype: classical vs atypical	Severe adverse reactions
Short term group									
1480	Female	4	2+2	0	2+2	c.622C>T p.(Arg208*)	c.1094G>A p.(Cys365Tyr)	Classical phenotype	no
1479	Female	3	2+2	0	2+1	c.509-1G>C	c.622C>T, p.(Arg208*)	Classical phenotype	Anaphylactic reaction to enzyme
1488	Male	4	1+1	0	1+1	c.622C>T p.(Arg208*)	c.1678_1679del	Classical phenotype	no
1498	Male	8	2+2	77	2+2	c.622C>T p.(Arg208*)	c.511G>C	Atypical phenotype	Infection with Initial infusion. Device removed and replaced.
1502	Female	4	2+2	0	2+2	c.509-1G>C	c.509-1G>C	Classical phenotype	no
1504	Male	4	2+2	0	2+2	c.379C>T p.(Arg127*)	c.509-1G>C	Classical phenotype	no
Long term group				**Months from first ERT to first Nfl measurement**					
1305	Male	4	2+0	9	1+0	c.1052G>T, p.Gly351Val	c.1052G>T, p.Gly351Val	Classical phenotype, Movement disorder and seizures settled over last 2 years	no
1306	Female	4	1+2	15	1+2	c.509-1G>C	c.509-1G>C	Classical phenotype, Severe movement disorder. Slight improvement observed with ERT	no
330	Male	5	1+1	15	0+0	c.509-1 G>C	c.509-1 G>C	Classical phenotype	no
331	Female	4	3+2	12	3+2	c.509-1 G>C	c.509-1 G>C		vomiting and myoclonic jerks after infusion
1128	Female	4	1+0	14	0+0	c.89+5G>A	c.509-1 G>C	Classical phenotype, Poorly controlled seizures	no
667	Female	15	2+2	12	2+2	c.89+5G>C	c.1340G>A p.(Arg447His)	Atypical phenotype	no

**Figure 1.**
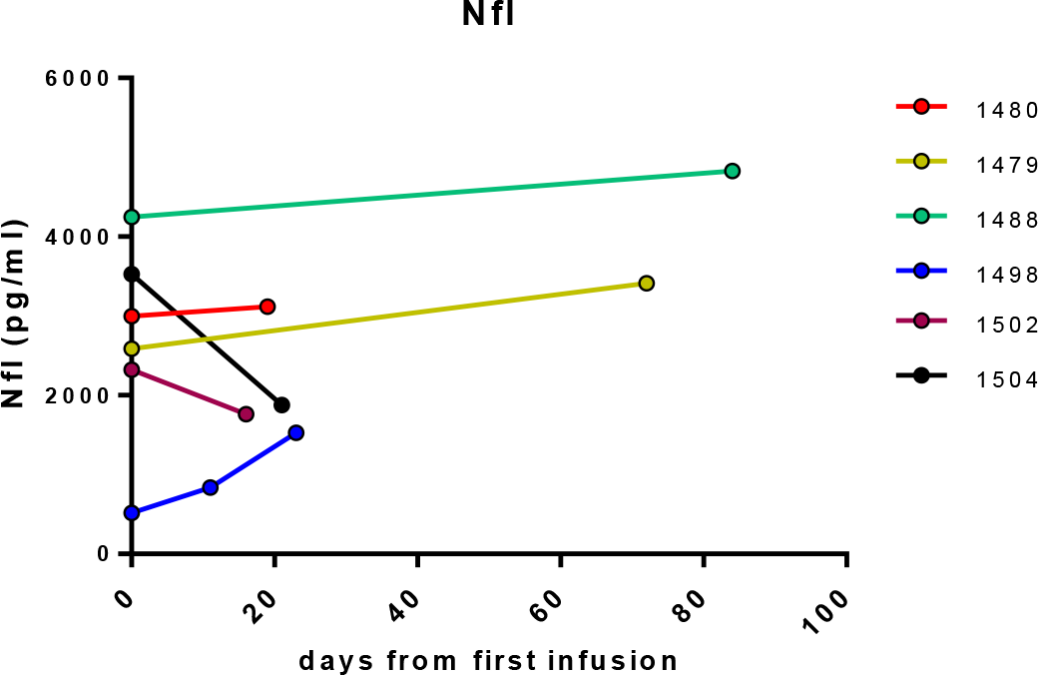
CSF Nfl levels (pg/ml) in CSF, showing response of patients in the short term group at start of treatment and followed up from 11-84 days after.

**Figure 2.**
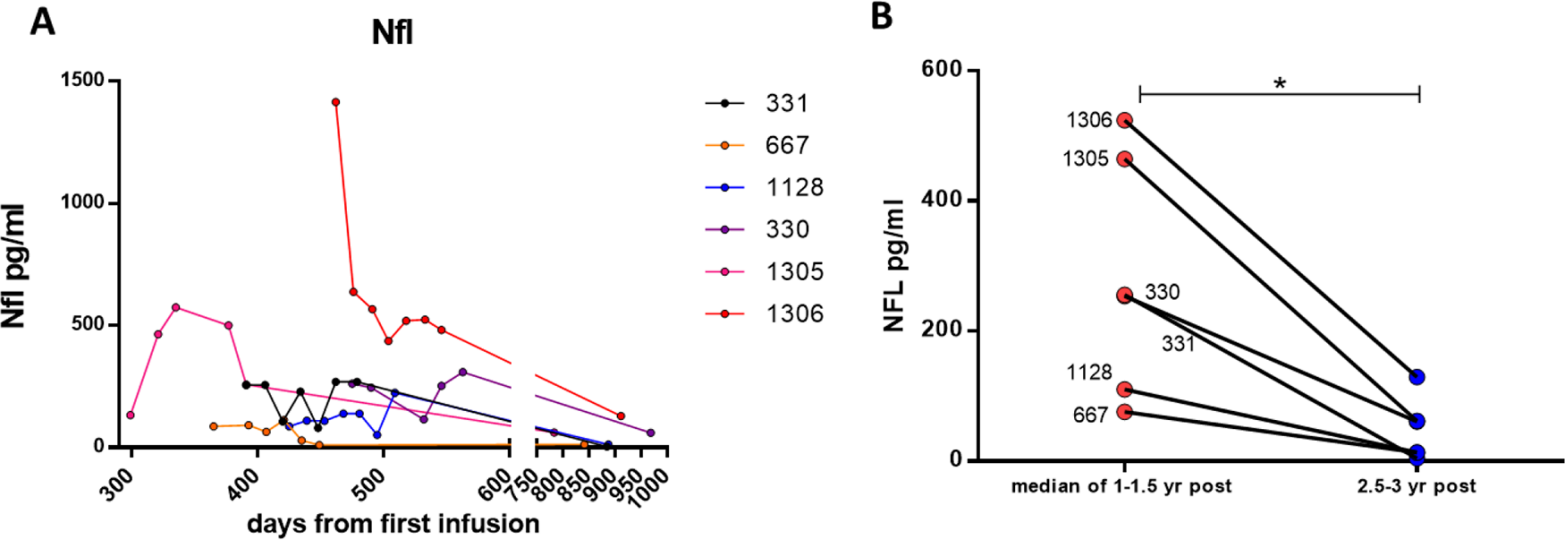
CSF NFL levels in the long term group (A) Longer term changes in a separate cohort of patients from 299 (9-10 months) to 911 days (~2.5 yrs) post treatment. (B) Paired analysis of the median values of NFL at 1-1.15 of treatment vs 2.5-3 yrs treatment. * *p* < 0.05 Wilcoxon paired t-test.

In the first cohort of “treatment-naïve” patients, the post-treatment samples collected 2-3 weeks after the first cerliponase alfa infusion showed that NfL had decreased in two patients ( Figure 1) but these values were still far higher than levels observed for patients on more than two years of ERT ( Figure 2A). The other three patients showed either no change or an increase in NfL.

In this cohort of six patients, the patient (1488) with the lowest combined language and motor CLN2 score had the highest NfL level. Patient 1498 has an atypical form of the disease with later onset of symptoms and much slower progression and had the lowest initial NfL levels in this cohort, but this increased after start of treatment. This increase is likely due to NfL levels being affected by an intracerebroventricular (ICV) device infection and repeat surgery in this patient, as NfL is a non-specific marker of neuroinflammation.

The second (long-term ERT) cohort started their treatment prior to approval of Brineura and had been receiving infusions as part of a compassionate use programme for a year before collection of CSF. For this group CSF began to be collected from 299 days (~10 months) to 600 days (~1.64 years) after the start of treatment and then continued to be collected up to 990 days (2.7 years) post treatment. Most patients had lower levels of NfL that were in the normal adult range (<300pg/ml), apart from two patients. These two patients (1305 and 1306) had more severe movement disorders compared with the other patients. Improvement in involuntary movements temporarily correlated with a decrease of NfL over the next 18 months. Patient 1128 has a particularly severe seizure disorder with poor response to pharmacotherapy. Their NfL level after one year of treatment was low suggesting that NfL level is not likely to be a useful biomarker of seizure control. This observation is supported by a previous study which reported that serum NfL was not altered in children with febrile seizures. ^12^ However, it had also previously been reported that NfL may reflect the contribution of seizure status to CLN3 disease severity. ^13^

The lowest NfL levels in this cohort was in the atypical patient 667. For the long-term ERT cohort, the one-year NfL levels fluctuated moderately before dropping to their lowest levels at two years after start of ERT. When taking the median value of 1-1.5 yrs post-ERT and comparing with levels >2.5 years after ERT there was a significant decrease in NfL levels ( *p* < 0.03 by paired t-test ( Figure 2B). This confirms previous observations where serum NfL levels were seen to continually decrease over three years on treatment. ^10^ That study also revealed that some patients’ early serum NfL levels (approximately two months post-ERT) increased and did not begin to decline until nearly one year on treatment. This previous study and our observations here for CSF NfL indicate that in some patients it may take up to a year to start seeing the positive effect of ERT on preventing axonal damage. Moreover, the complete normalisation of NfL levels is likely to take longer. Once the levels normalise, they are likely to stay in the normal range which corresponds to the stabilisation of the patients’ clinical parameters.

## Conclusions

CSF NfL levels are increased in CLN2 disease patients with lower levels observed in patients with an atypical phenotype therefore Nfl levels potentially indicate disease progression and show the effect of treatment with Brineura, correlating with the decline of neuroaxonal damage to very low levels after 2.8 years of ERT. However, in some patients we observed a delayed decline to low levels in Nfl compared to other CLN2 patients. CSF NfL may therefore be a good marker to identify these patients who could then receive an adjusted treatment regimen to achieve a faster improved clinical outcome. The results reported here are also relevant to interpretation of NfL changes with time in a clinical trial of potentially disease-modifying drug candidates in adult neurodegeneration. ^14^

## Data availability

### Underlying data

UCL data repository: CSF Nfl results CLN2 disease. https://doi.org/10.5522/04/14822463.v1. ^15^

The project contains CSF Nfl results for Group 1 and Group 2 (CSF Nfl CLN2 disease Iwan etal.xlsx).

Data are available under the terms of the Creative Commons Zero “No rights reserved” data waiver (CC0 1.0 Public domain dedication).
