# Supplementary material for: Cerebrospinal fluid neurofilament light chain levels in CLN2 disease patients treated with enzyme replacement therapy normalise after two years on treatment
Source: F1000Res. 2022 Jan 5;10:614. Originally published 2021 Jul 20. [Version 2] doi: 10.12688/f1000research.54556.2 (PMC8777495; doi:10.12688/f1000research.54556.2)
Supplement: Peer review history

# Peer review: Reviewer response for version 2

**DOI:** 10.5256/f1000research.80101.r118695

**Published:** 2022-01-20

**Authors:** Michel Boutin

**Affiliations:** 1 Division of Medical Genetics, Department of Pediatrics, Centre de Recherche-CHUS, Faculty of Medicine and Health Sciences, Université de Sherbrooke, Sherbrooke, QC, Canada

All the corrections that I requested were done. So I approve the latest version of the manuscript without reservations.

Is the work clearly and accurately presented and does it cite the current literature?

Yes

If applicable, is the statistical analysis and its interpretation appropriate?

Yes

Are all the source data underlying the results available to ensure full reproducibility?

Yes

Is the study design appropriate and is the work technically sound?

Yes

Are the conclusions drawn adequately supported by the results?

Yes

Are sufficient details of methods and analysis provided to allow replication by others?

Yes

Reviewer Expertise:

I have a Ph.D. in chemistry and I performed postdoctoral trainings in proteomics and metabolomics. Since 2011, I am the technical director of the Waters-CHUS Expertise Centre in Clinical Mass Spectrometry (Sherbrooke, QC, Canada). My main area of research concerns the discovery of biomarkers for rare genetic diseases using untargeted metabolomics.

I confirm that I have read this submission and believe that I have an appropriate level of expertise to confirm that it is of an acceptable scientific standard.

# Peer review: Reviewer response for version 1

**DOI:** 10.5256/f1000research.58055.r96195

**Published:** 2021-11-09

**Authors:** Daniel Erskine

**Affiliations:** 1 Translational and Clinical Research Institute, Newcastle University, Newcastle upon Tyne, UK

This manuscript, by Iwan and colleagues, set out to longitudinally follow patients with the CLN2 form of Neuronal Ceroid Lipofuscinosis, to detect response to enzyme replacement therapy by evaluating CSF levels of NfL. There is a pressing need to better evaluate response to treatment in CLN2 patients receiving enzyme replacement therapy, as current measures are limited to clinical instruments. Whilst clinical scales are useful to show functional improvement, clinical features likely follow from progressive degeneration over time, and thus may lack the sensitivity to detect changes in neurodegeneration. Evaluation of NfL levels in CSF has been gaining traction as a relatively broad measure of axonal degeneration across a range of neurodegenerative conditions, and the authors quite reasonably suggest this may be a more sensitive measure of treatment response compared to clinical tests alone. NfL levels were measured in CSF using the highly sensitive SIMOA platform that is optimised for detecting even low levels of the analyte of interest. They identified a reduction in NfL over time in most cases following enzyme replacement therapy, but this was often 2-3 years following treatment initiation. The authors note the variability in response and discuss the potential of using this information to monitor treatment response and potentially adjust regimens.

 Overall, I thought this was an excellent manuscript. Given the rarity of CLN2 and the longitudinal nature of this study, the number of participants was particularly impressive. I have some suggestions for the authors to consider:

- I felt there was a lack of relationship with clinical variables. Given that the stated aim of the study was to augment current monitoring with CLN2 CRS, I was surprised to see no attempt at associating changes in CLN2 CRS with NfL levels over time.
- Was any imaging performed to corroborate the suggestion that changes in NfL are associated with reduced axonal degeneration or neurodegeneration?

Is the work clearly and accurately presented and does it cite the current literature?

Yes

If applicable, is the statistical analysis and its interpretation appropriate?

Yes

Are all the source data underlying the results available to ensure full reproducibility?

Yes

Is the study design appropriate and is the work technically sound?

Yes

Are the conclusions drawn adequately supported by the results?

Yes

Are sufficient details of methods and analysis provided to allow replication by others?

Yes

Reviewer Expertise:

Neurodegenerative disease pathology, neurometabolic disease pathology

I confirm that I have read this submission and believe that I have an appropriate level of expertise to confirm that it is of an acceptable scientific standard.

# Response

**Published:** 2021-12-03

**Authors:** Wendy Heywood

**Affiliations:** UCL, UK

*Many thanks for reviewing the report.*

*We tried to analyse whether the speed of loss of points on CLN2 clinical score is correlated with the slope of NFL normalisation, i.e. whether NFL levels can act as a biomarker of response to enzyme replacement therapy (ERT). We did indeed look at this and did not find a statistically significant correlation. This could be due to a few factors such as the small number of patients in the study, the fact that CLN2 score is not sufficiently sensitive to detect small changes in response or there is no real correlation between the NFL levels and the patients response to ERT.*

*In response ot your other question the MRI scans are performed yearly in the patients which is similar to the clinical trial previously reported by Schulz et al. NEJM 2018, where MRI grey matter volume changes was one of the outcome measures. The results in our patients were in line with the clinical trial report where patients lost approximately 10.5% of total grey matter volume in the first year of treatment and 3.3%in the second year which indeed indicates neurodegenerative processes.*

# Peer review: Reviewer response for version 1

**DOI:** 10.5256/f1000research.58055.r90536

**Published:** 2021-08-11

**Authors:** Michel Boutin

**Affiliations:** 1 Division of Medical Genetics, Department of Pediatrics, Centre de Recherche-CHUS, Faculty of Medicine and Health Sciences, Université de Sherbrooke, Sherbrooke, QC, Canada

The authors of this brief report have tested the possibility to use the neurofilament light chain (NfL) levels in cerebrospinal fluid as a biomarker to monitor the response to cerliponase alfa for patients suffering from CLN2, the classic late infantile neuronal ceroid lipofuscinosis. The biomarkers were analysed in specimens of six patients at the initiation of treatment and followed up from 14-84 days after, and for six other patients from 9-10 months to 2.5 yrs post-treatment. Since CLN2 is an extremely rare genetic disease, I think that it was a major accomplishment to longitudinally monitor the biomarkers for a cohort of 12 patients. The results obtained revealed a statistically significant long-term decrease of NfL levels in cerebrospinal fluid of CLN2 patients treated with cerliponase alfa. However, soon after the initiation of ERT (≤ 84 days), the biomarker levels increased for some patients and decreased for others. It suggests that the NfL levels might be used to adjust patient regimen, but further studies will be needed to confirm it. Moreover, we must keep in mind that NfL is a non-specific marker of neuroinflammation, and its level is also affected by repeat surgeries and infections due to the intracerebroventricular device used to dispense the enzyme replacement therapy.

 Minor revisions:

1.  In the title, I suggest adding “chain” between “light” and “levels”.
2. In the second paragraph of the “Samples” section, I suggest adding the limit of detection and the limit of quantification of the Simoa kit, as well as the concentration of the internal quality control sample.
3. In the “Analysis section”, I suggest replacing “Results were exported to Microsoft Excel ^15^” with “Results were downloaded in the UCL data repository ^15^”

1. I think that it can be less confusing for the reader if the patients are labeled 1 to 12, instead of 1480, 1479, 1488, 330 … in the figures, the table, and in the core of the manuscript.
2. In the "Results and Discussion" section, second paragraph, first line: Please replace (n = 5) by (n = 6).
3. For Figures 1 and 2, the shape of the data point marks might be changed for the different patients (Ex: square, triangle ...) to facilitate the comprehension of the graphs when the article is printed in black and white.

Is the work clearly and accurately presented and does it cite the current literature?

Yes

If applicable, is the statistical analysis and its interpretation appropriate?

Yes

Are all the source data underlying the results available to ensure full reproducibility?

Yes

Is the study design appropriate and is the work technically sound?

Yes

Are the conclusions drawn adequately supported by the results?

Yes

Are sufficient details of methods and analysis provided to allow replication by others?

Yes

Reviewer Expertise:

I have a Ph.D. in chemistry and I performed postdoctoral trainings in proteomics and metabolomics. Since 2011, I am the technical director of the Waters-CHUS Expertise Centre in Clinical Mass Spectrometry (Sherbrooke, QC, Canada). My main area of research concerns the discovery of biomarkers for rare genetic diseases using untargeted metabolomics.

I confirm that I have read this submission and believe that I have an appropriate level of expertise to confirm that it is of an acceptable scientific standard, however I have significant reservations, as outlined above.

# Response

**Published:** 2021-12-03

**Authors:** Wendy Heywood

**Affiliations:** UCL, UK

Many thanks. All the recommended changes have been made to the manuscript.
